# 2421: Patient and household member colonization and environmental contamination with *Staphylococcus aureus* in a comparative effectiveness study of home-based interventions to reduce CA-MRSA recurrence and household transmission

**DOI:** 10.1017/cts.2017.273

**Published:** 2018-05-10

**Authors:** Jonathan N. Tobin, Rhonda G. Kost, Brianna M. D’Orazio, Chamanara Khalida, Jessica Ramachandran, Mina Pastagia, Teresa H. Evering, Maria Pardos de la Gandara, Cameron Coffran, Joel Correa da Rosa, Kimberly Vasquez, Getaw Worku Hassen, Tracie Urba

## Abstract

OBJECTIVES/SPECIFIC AIMS: Community-associated methicillin-resistant *Staphylococcus aureus* (CA-MRSA) skin and soft tissue infections (SSTIs) are commonly seen in primary care, with recurrence rates that range from 16% to 43%, and present significant challenges to clinicians, patients, and families. This comparative effectiveness research study aims to develop and evaluate a home-based intervention implemented by Community Health Workers (CHWs) or “promotoras” to prevent recurrence of CA-MRSA in patients presenting to primary care with SSTIs and transmission within their households. This presentation will examine associations between wound microbiology, clinical presentation, and housing characteristics, including housing density and household surfaces contamination. METHODS/STUDY POPULATION: In partnership with 3 Community Health Centers and 3 community hospitals in NYC, this study will recruit patients (n=278) with confirmed MRSA SSTIs and their household members. Participants will be randomized to receive either a CHW/Promotora-delivered decolonization-decontamination intervention (based on the REDUCE MRSA trial) or usual care. The highly engaged stakeholder team finalized the intervention protocol, developed and implemented CHW and clinician training, and developed an online health portal application for data management and exchange. RESULTS/ANTICIPATED RESULTS: We have collected 923 isolates from 237 individuals, including 240 wound culture isolates and 683 surveillance culture isolates (nares, axilla, groin). MRSA and MSSA were found in 19% and 21.1% of wound cultures, respectively; 59.5% with MRSA+ wound culture had 1 or more MRSA+ surveillance culture; 67.8% with MSSA+ wound culture had 1 or more MSSA+ surveillance culture. Of those with MRSA or MSSA infections, 70% of subjects were male, with an average age of 37.9 (SD=15.9 y). The most frequent sites of infection were the leg (20%), axilla (18%), buttock (17%), and abdomen/torso (12%). There was no association between the location and type of infection (MRSA/MSSA) (*p*-value=0.09). The kitchen floor (14.05%) and bedroom floor (14%) were the most common surfaces contaminated with MRSA. These were also the most common surfaces contaminated with MSSA, which was recovered from 10.2% and 9.1% of kitchen floors and bedroom floors, respectively. For individuals with an MRSA or MSSA wound infection, there was an average number of 3.2 (SD=1.6) co-residents per household, and 36.5% of household members were colonized with either MRSA or MSSA. There is no association between household density (number of co-residents) and type of infection (MRSA/MSSA) (Fisher’s *p*-values=0.171 and 0.371, respectively). In households of participants with MSSA wound infections, the number of colonized sites is positively associated with the level of household MSSA contamination (*p*=0.027). Further analyses will examine the associations between molecular subtypes, wound location, household surface contamination and household member colonization and infection. DISCUSSION/SIGNIFICANCE OF IMPACT: This study aims to understand the patient-level and environmental-level factors associated with SSTI recurrence, surface contamination and household transmission, and to examine the interactions between bacterial genotypic and clinical/phenotypic factors on decontamination, decolonization, SSTI recurrence and household transmission. This study will evaluate the barriers and facilitators to implementation of home visits by CHWs in underserved populations, and aims to strengthen the evidence base for implementation of strategies to identify and reduce household reservoirs and then control SSTI recurrence and household transmission.

